# Should rapid antigen tests be first-line for COVID-19 testing? Results of a prospective urban cohort study

**DOI:** 10.1186/s12879-023-08171-6

**Published:** 2023-04-18

**Authors:** Mohamad Rani Hassoun, Nathan T. Kudlapur, Grace M. Chen, Jenna Green-Ross, Ashlesha Patel

**Affiliations:** 1 Feinberg School of Medicine, Northwestern University, Chicago, IL USA; 2 A.T. Still University School of Osteopathic Medicine in Arizona, Mesa, AZ USA; 3 John H. Stroger, Jr. Hospital of Cook County, Chicago, IL USA

**Keywords:** COVID-19, Rapid antigen test, SARS-CoV-2, Diagnostic accuracy, Validation study

## Abstract

**Background:**

A highly accurate, rapid, and low-cost COVID-19 test is essential for guiding isolation measures. To date, the most widely used tests are either nucleic acid amplification tests or antigen tests. The objective of this study is to further assess the diagnostic performance of the Binax-CoV2 rapid antigen test in comparison to the current gold standard reverse transcription quantitative polymerase chain reaction (RT-qPCR), with additional analysis of symptomatology and cycle threshold utility.

**Methods:**

This is a prospective cohort study performed between November and December 2020. Individuals who presented to COVID-19 testing events and received both RT-qPCR and a rapid antigent test were included. Testing occurred at the emergency department of an urban hospital and at a community mobile unit. No fees or appointments were required. Individuals self-reported the presence or absence of symptoms and history of positive COVID-19 test within the previous two weeks. Trained staff collected two subsequent nasopharyngeal swabs of both nares. One set of swabs underwent RT-qPCR and the other underwent Binax-CoV2 assay per manufacturer guidelines.

**Results:**

A total of 390 patients were included, of which 302 were from the community site. Of these 302, 42 (14%) were RT-qPCR positive. Of the 42 RT-qPCR positive, 30 (71.4%) were also positive by Binax-CoV2. The Binax-CoV2 test had a sensitivity of 71.4% (95% CI: 55%–84%) and a specificity of 99.6% (95% CI: 98%–100%) in this population. Performance of the Binax-CoV2 test performed better in individuals with higher viral load. For symptomatic patients with cycle threshold < 20, sensitivity reached 100%.

**Conclusions:**

The Binax-CoV2 assay’s specificity and sensitivity in individuals with high viral load makes it a suitable first-line test for detecting COVID-19. However, given the assay’s measured sensitivity, a negative result on the Binax-CoV2 assay may warrant additional testing with more sensitive tests, such as the RT-qPCR. This is particularly the case with high clinical suspicion for an active SARS-CoV-2 infection even after a negative Binax-CoV2 result.

## Introduction

For the past few years, severe acute respiratory syndrome coronavirus 2 (SARS-CoV-2), the causative agent of COVID-19, has propagated rapidly throughout the United States, taking with it over a million lives [1]. Since then, the number of COVID-19 cases, hospitalizations, and deaths have plummeted significantly in the United States, due in large part to public health measures and widespread vaccination efforts. However, the virus has continued to cause intermittent outbreaks around the world, and it has become evident that eradication of the virus is unlikely [2]. Given this, it becomes apparent that having a test that is able to quickly and accurately detect individuals with an active SARS-CoV-2 infection will prove to be essential and beneficial for years to come.

To detect an infection, a nucleic acid amplification test (NAAT), such as reverse transcription quantitative polymerase chain reaction (RT-qPCR), or an antigen test is performed. RT-qPCR works by detecting viral RNA in a respiratory sample, such as a nasal swab. To date, NAATs are considered the gold standard for detecting SARS-CoV-2 infections [3]. NAATs, such as RT-qPCR, have their share of pros and cons. For instance, given its highly sensitive nature, RT-qPCR will not only give positive results for those with an active SARS-CoV-2 infection, but it may presumably even give positive results for a long period of time thereafter [4]. More specifically, even after an individual is deemed noninfectious by public health guidelines, one may remain RT-qPCR positive for 3 months [5]. RT-qPCR therefore may not be an optimal indicator of infectivity, particularly when an individual is asymptomatic or remotely symptomatic. Moreover, RT-qPCR generally has a turnaround time of more than 24 hours to perform, is expensive, and requires highly trained personnel [6]. Given this, RT-qPCR is somewhat limited in its utility in the acute setting, such as in an emergency department where it must be quickly decided which patients need to be isolated from others.

An ideal COVID-19 test is one with a quick turnaround time, is inexpensive, does not require much technical expertise, and yet is accurate. As of this writing, there are more than 1,000 diagnostic tests commercially available worldwide, with varying test parameters such as target analyte and sample types [7]. One example of such a test is the Abbott BinaxNOW™ COVID-19 Ag Card (hereafter referred to as Binax-CoV2), a lateral flow immunoassay whose target antigen is the N-protein. The Binax-CoV2 test received Emergency Use Authorization (EUA) from the Food & Drug Administration (FDA) in 2020 [8]. The Binax-CoV-2 test is listed as having a turnaround time of under 30 min, a retail price of under $15, and can be completed at home without additional expertise needed. In this study, we seek to characterize the performance characteristics of the Binax-CoV2 test.

Previous studies have reported high values for the sensitivity and specificity of the Binax-CoV2 test [9–11]. Of note, there is a lack of such studies conducted here in the Midwest. The primary aim of this study was to further evaluate the results of the Abbott BinaxNOW™ COVID-19 Ag Card in comparison to the gold standard RT-qPCR in a different geographic population at two urban testing sites in the Midwest. Moreover, we sought to evaluate whether the presence or absence of symptoms impacted the utility of the rapid test. Because rapid antigen tests do not have an amplification step, we hypothesize that the Abbott BinaxNOW™ COVID-19 Ag Card will have more accurate results in individuals with higher viral loads and who are symptomatic than in individuals without symptoms and with lower viral loads, giving more false negatives in these latter individuals.

## Methods

This prospective convenience cohort study was performed at two different testing sites in the city of Chicago on three separate days. The study was exempt from Institutional Review Board approval, as it was a quality assurance project to assess the accuracy of a rapid COVID-19 test. The study adhered to the tenets of the Declaration of Helsinki and the regulations of the Health Insurance Portability and Accountability Act of 1996.

### Study design

COVID-19 testing events were held on three days in November and December of 2020. The first event was at a mobile testing site located near a large mall in Calumet City, IL, and the second and third testing events were located in the emergency department (ED) of John H. Stroger, Jr. Hospital of Cook County in Chicago, IL. An appointment was not needed to get tested, and anyone was able to get tested. Testing was free of charge for participants without insurance. Upon arrival, participants were asked to self-report demographic information, insurance type, presence or absence of symptoms, and whether they had recently been in known contact with an individual with active COVID-19. During the first ED event, the screening questionnaire included an item asking whether the patient had received a prior positive COVID-19 test within the past two weeks. Only the patients who provided informed consent to receive both the PCR and Binax-CoV2 tests were included in this validation study.

### Specimen collection and processing

Two subsequent nasopharyngeal (NP) swabs of both nares were performed by trained staff, one set being for Binax-CoV2 testing and the other for RT-qPCR testing. The Binax-CoV2 assay was completed on-site, as described by the manufacturer [8], and results were read by trained staff. The specimens designated for RT-qPCR were taken back to John H. Stroger, Jr. Hospital of Cook County. RT-qPCR was then performed using either the Abbott RealTime SARS-CoV-2 assay on the Abbott m2000 system or the Panther Fusion® SARS-CoV-2 assay on the Panther system, as previously described by the respective manufacturers [12, 13]. The Abbott RealTime SARS-CoV-2 assay is a dual target assay for the RdRp and N genes, and the Panther Fusion® SARS-CoV-2 assay is an assay for the ORF1ab gene. Participants were informed of their RT-qPCR results, but their Binax-CoV2 results were not disclosed, as it was a validation test.

### Analysis

Statistical analysis was performed using SPSS Statistics version 26 and Microsoft Excel. Several test parameters were calculated, as described below. Of note, the sensitivity and specificity analyzed in this manuscript were clinical parameters. Sensitivity is defined here as the ratio of samples with a positive Binax-Cov2 and a positive RT-qPCR to all samples with a positive RT-qPCR. Specificity was defined as the ratio of samples with a negative Binax-Cov2 and a negative RT-qPCR to all samples with a negative RT-qPCR. Likewise, positive predictive value (PPV) was defined here as the ratio of samples with a positive Binax-CoV2 and positive RT-qPCR to all those with a positive Binax-CoV2. Negative predictive value (NPV) was defined as the ratio of samples with a negative Binax-CoV2 and negative RT-qPCR to all those with a negative Binax-CoV2.

## Results

### Comparison of RT-qPCR and Binax-CoV2 in the community setting

At the community mobile unit testing site, a total of 302 patients were tested for COVID-19 with both RT-qPCR and Binax-CoV2. Self-reported patient characteristics are listed in Table 1. Of these individuals, 42 (14%) were RT-qPCR–positive. Of the 42 RT-qPCR positive patients, 30 were also positive for the Binax-CoV2 test. Fourteen (33%) of the RT-qPCR positive patients were asymptomatic. With a prevalence of 14%, the Binax-CoV2 test performed with a sensitivity of 71.4% (95% confidence interval [CI], 55%–84%) and a specificity of 99.6% (95% CI, 98%–100%) (Table 2). This corresponds to a PPV of 0.97 (0.83–1.00) and a NPV of 0.96 (0.92–0.98). In symptomatic patients, performance of the Binax-CoV2 test translated to 79% sensitivity, 98% specificity, 0.96 PPV, and 0.90 NPV, in comparison to the asymptomatic group with 57% sensitivity, 100% specificity, 1.00 PPV and 0.77 NPV.

**Table 1:** Self-reported characteristics of patients tested in the community and emergency department setting

	**Community Setting**	**Emergency Department Setting**
**Characteristic**	**n** (%)	**n** (%)
Total	302 (100)	88 (100)
Age Group (Years)		
≤18	36 (12)	1 (<1)
19-34	42 (14)	19 (22)
35-49	85 (28)	24 (27)
50-64	60 (20)	33 (38)
≥65	79 (26)	11 (13)
Gender		
Female	160 (53)	34 (39)
Male	142 (47)	54 (61)
Race		
White	172 (57)	—
African American / Black	69 (23)	—
Asian	15 (5)	—
American Indian / Alaskan Native	6 (2)	—
Native Hawaiian / Pacific Islander	1 (<1)	—
Other	39 (13)	—
Ethnicity		
Hispanic / Latino / Spanish Origin	24 (8)	—
Non-Hispanic / Latino / Spanish Origin	278 (92)	—
Insurance Type		
Self-Pay	175 (58)	—
Private	97 (32)	—
Public	30 (10)	—
Symptomatology		
Symptomatic	84 (28)	15 (17)
Asymptomatic	218 (72)	73 (83)

Reported characteristics at the community setting include age, gender, race, ethnicity, insurance type, and presence or absence of symptoms. Reported characteristics at the emergency department setting include age, gender, and presence or absence of symptoms

**Table 2:** Binax-CoV2 performance characteristics in the community setting

	**Community Department Setting**								
	**Binax-CoV2 Result**	**RT-PCR (+) Patients**	**RT-PCR (-) Patients**	**Prevalence**	**Sensitivity (95% CI)**	**Specificity (95% CI)**	**PPV**	**NPV**	**Total**
All patients	Positive	30	1	0.14	0.71 (0.55–0.84)	0.996 (0.98–1.00)	0.97 (0.83–1.00)	0.96 (0.92–0.98)	31
	Negative	12	259	–	–	–	–	–	271
Total		42	260						302
Symptomatic patients	Positive	22	1	0.33	0.79 (0.59–0.92)	0.98 (0.90–1.00)	0.96 (0.78–1.00)	0.90 (0.80–0.96)	23
	Negative	6	55	–	–	–	–	–	61
Total		28	56						84
Asymptomatic patients	Positive	8	0	0.06	0.57 (0.29–0.82)	1.00 (0.98–1.00)	1.00 (0.63–1.00)	0.97 (0.94–0.99)	8
	Negative	6	204	–	–	–	–	–	210
Total		14	204						218

*Abbreviations*: *Binax-CoV2* Abbott BinaxNOW™ COVID-19 Ag Card, *RT-qPCR* reverse transcription quantitative polymerase chain reaction, *CI* confidence interval, *PPV* positive predictive value, *NPV* negative predictive value

**Fig. 1:**
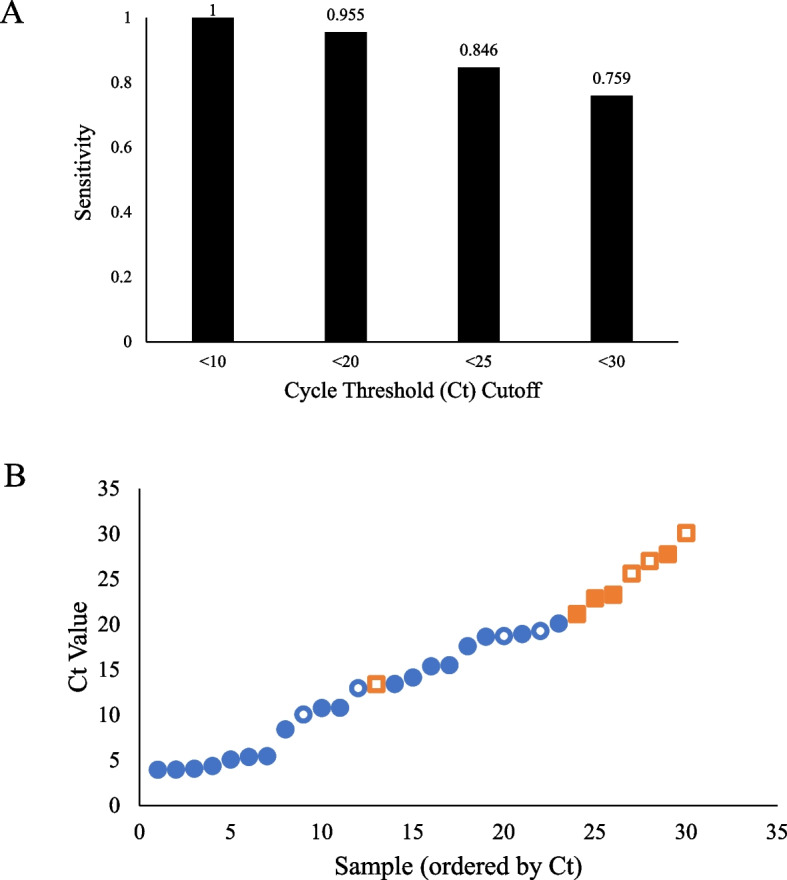
Binax-CoV2 performance based on Cycle Threshold (Ct) cutoffs. **A** Bar graphs represent the sensitivity of the Binax-CoV2 assay at various Ct thresholds. Performance of the assay is optimized at Ct < 20, where sensitivity is > 95%. **B** Ct value of all patient samples from the community setting included in the Ct analysis, plotted in ascending order. Blue circles represent Binax-CoV2–positive samples; orange squares represent Binax-CoV2–negative samples. Asymptomatic patients are indicated by open symbols, and symptomatic patients are indicated by filled symbols

Of the 42 RT-qPCR positive patients, 30 were tested with the Abbott RT-qPCR test and 12 were tested with the Hologic RT-qPCR test. The Abbott test provides data on cycle threshold (Ct) values, but Hologic testing does not. Therefore, based on data with Abbott testing, we stratified Binax-CoV2 results by Ct value (Fig. 1A). When taking patients only with Ct < 20, sensitivity of Binax-CoV2 improves to 95.5%. Furthermore, taking patients that are both symptomatic and have Ct < 20, the Binax-CoV2 test reaches 100% sensitivity.

### Comparison of RT-qPCR and Binax-CoV2 in the emergency department setting

 A total of 88 patients were tested for COVID-19 in the ED on two different testing dates, with both RT-qPCR and Binax-CoV2. Self-reported patient characteristics are listed in Table 1. Of these, 17 (19%) individuals were RT-qPCR positive, and 10 were Binax-CoV2–positive (Table 3). Thus, with a prevalence of 19%, the Binax-CoV2 test performed with 53% sensitivity and 99% specificity in the ED setting. Analysis was also performed based on symptomatology. Binax-CoV2 sensitivity was 60% for symptomatic patients compared to 38% for asymptomatic patients. Since patients from the first ED event provided information regarding positive COVID-19 tests in the past two weeks, we further stratified this subset of patients. After removing patients that had tested positive for COVID-19 in the two weeks prior, sensitivity of Binax-CoV2 improved from 43 to 71%. As patients in the ED were all tested with Hologic RT-qPCR, data was not available to perform Ct-based analysis.

**Table 3:** Binax-CoV2 performance characteristics in the emergency department setting

	**Emergency Department Setting**								
	**Binax-CoV2 Result**	**RT-PCR (+) Patients**	**RT-PCR (-) Patients**	**Prevalence**	**Sensitivity**	**Specificity**	**PPV**	**NPV**	**Total**
All patients	Positive	9	1	0.19	0.53	0.99	0.90	0.90	10
	Negative	8	70	–	–	–	–	–	78
Total		17	71						88
Symptomatic patients	Positive	3	0	0.33	0.60	1.00	1.00	0.83	3
	Negative	2	10	–	–	–	–	–	12
Total		5	10						15
Asymptomatic patients	Positive	6	0	0.22	0.38	1.00	1.00	0.85	6
	Negative	10	57	–	–	–	–	–	67
Total		16	57						73
ED Event 1	Positive	6	1	0.29	0.43	0.97	0.86	0.80	7
	Negative	8	33	–	–	–	–	–	41
Total		14	34						48
ED Event 1^a^	Positive	5	1	0.18	0.71	0.97	0.83	0.94	6
	Negative	2	31	–	–	–	–	–	33
Total		7	32						39

*Abbreviations*: *Binax-CoV2* Abbott BinaxNOW™ COVID-19 Ag Card, *RT-qPCR* reverse transcription quantitative polymerase chain reaction, *CI* confidence interval, *PPV* positive predictive value, *NPV* Negative predictive value

^a^Excluding patients that had a prior positive COVID-19 test in the previous 2 weeks

## Discussion

The COVID-19 pandemic has posed a major challenge in the need to rapidly identify cases in order to inform quarantine and isolation practices. Current gold standard RT-qPCR tests may remain positive for up to 3 months after an original diagnosis [14]. This phenomenon creates challenges in directing isolation measures when individuals require a negative test to return to work or other activities. However, as seen here and elsewhere, rapid antigen tests maintain acceptable levels of sensitivity and specificity while also correlating with Ct values, a marker for infectivity.

A number of studies have correlated Ct values with infectivity of SARS-CoV-2 [15–17]. Since Ct refers to the number of RT-qPCR cycles required to exceed background levels of detectable signal, higher Ct values are associated with lower viral load, which translates to less infectivity and lower risk of transmission. In the hospital setting, patients with a Ct value above 33 have been deemed noncontagious [17]. Another study focused on a community setting demonstrated a Ct value of 30 as the threshold for transmissibility [9].

In this study, we report a Ct cutoff of 20 for optimal performance of the Binax-CoV2 assay. We chose this more conservative threshold as it correlates with a sensitivity of > 95%. As demonstrated in Fig. 1B, a Ct of 20 is the threshold at which our data demonstrate a transition to false negative results as reported by the assay. Furthermore, patients are more likely to present with symptoms at Ct < 20 vs Ct > 20 (78% vs 57%, respectively). Thus, a cutoff of Ct 20 better aligns with clinical suspicion deriving from symptomatic presentation. Although we report a Ct cutoff of 20 for optimal performance, it is true that individuals with Ct up to 30 may have transmissible virus. However, the probability of transmission is likely lower in individuals with a higher Ct because of suspected lower viral load. Our data demonstrate that the Binax-CoV2 assay is highly unlikely to yield a false negative result in patients with Ct < 20. Thus, the questionable territory is when a negative Binax-CoV2 results in a sample that has Ct > 20. In these cases, a PCR test may be needed to confirm, and higher suspicion is warranted if the patient is symptomatic. Nevertheless, at Ct < 20 one can have high confidence in the test results.

Other studies examining the Panbio COVID-19 test (another commercially available rapid antigen test) report a similar Ct threshold for optimal performance [18–20]. In a study by Pilarowski et al., the authors reported a sensitivity of 93.3% for the Binax-CoV2 assay at a Ct < 30 [9]. At the same Ct cutoff in our study, sensitivity was only 75.9%. This difference could be attributed to multiple factors. First, sample size in our study (302) was about one-third that of Pilarowski et al.’s (878). Next, our study population consisted of a greater fraction of symptomatic individuals (28% vs 16%). This could suggest that patients in our sample were more susceptible to clinical display of SARS-CoV-2 infection, given that the Binax-CoV2 was less accurate in ruling out infection at lower viral loads. In a study by Kuo et al., the authors report a positive percent agreement (PPA) of 47% for the Binax-CoV2 test with two different RT-qPCR tests [21]. However, the study also shows that PPA was 100% for samples at Ct < 25. When accounting for dilutional effects of the samples, the authors predict PPA improves up to Ct 28–29.5. This is in agreement with the findings of our study that Binax-CoV2 performs better at higher viral loads and thus lower Ct thresholds. Higher Ct cutoffs in the Kuo et al. study could possibly be due to the fact that samples were taken from NP swabs, mid-turbinate nasal swabs, and anterior nasal swabs, whereas our study consisted of samples solely from NP swabs. The greater selection of swabs in Kuo’s study likely provided more samples with lower viral load, thereby necessitating a higher Ct value.

Given that these rapid antigen tests perform best in the setting of lower Ct and higher viral load, they will be most effective for informing clinicians of whether a patient is actively shedding the virus. This was also demonstrated by our data from the first ED testing event. After removing patients that had received a prior positive COVID-19 test, sensitivity of the Binax-CoV2 improved drastically. This is mainly due to the removal of patients that were testing negative on the Binax-CoV2 test but were still PCR positive. We thus pose the question of whether rapid antigen tests should be considered first-line in the diagnosis of COVID-19. The results of rapid antigen tests provide more reliable results on the infectious status of the individual. If the results of the diagnostic test will inform quarantine protocols, then infectivity should be the main consideration rather than the mere presence or absence of the virus. Thus, the test is not simply sacrificing accuracy for speed and cost but rather providing meaningful insight on the test result itself. For example, patients who recovered from COVID-19 may test positive on RT-qPCR tests for months afterward [22]. Even though there is no longer active, clinically infectious virus present, the RT-qPCR test may still report a positive result. However, a rapid antigen test is much more likely to report a negative result in this instance. Therefore, rapid antigen tests better predict our course of clinical action and should be considered for first-line use in the diagnosis of SARS-CoV-2 infection. They do not simply sacrifice but rather better demonstrate clinical parameters such as infectivity.

Based on these findings, we propose that rapid antigen tests such as the Binax-CoV2 assay should be utilized as first-line testing for COVID-19. If the test is positive, it is quite likely a true positive, so standard isolation measures should be taken. If the test is negative, however, then contextual information such as recent exposures and the presence of symptoms should be used in combination with a subsequent RT-qPCR test. This is supported by the findings in our study that in both the community and ED setting, Binax-CoV2 sensitivity was greater in the symptomatic cohort. This not only helps to streamline testing but also yields cost benefit. Further studies on rapid antigen tests could examine cost models to explore this impact.

Despite the findings of this study, a number of limitations must be recognized. First, as the Binax-CoV2 assay is examined by visual interpretation, results may be subjective. Second, not all patients who were Binax-CoV2–positive had Ct data due to some patients receiving the Hologic RT-qPCR test. This could potentially bias the data. Next, the symptom questionnaires did not report days between symptom onset and testing. This has the potential to be a confounding variable, as a shorter duration between symptom onset and testing would be more likely to yield both a positive Binax-CoV2 and RT-qPCR result. Finally, within the emergency department setting, the available sample size was modest.

As new variants continue to emerge, and as the pandemic continues to evolve, the utility and accuracy of such rapid tests will continue to be discussed. As such, new studies examining the clinical parameters with new variants and diverse populations would certainly be warranted.

## Data Availability

All data generated or analyzed during this study are included in this published article.

## Abbreviations

SARS-CoV2: Severe acute respiratory syndrome coronavirus 2
COVID-19: Coronavirus disease 2019
NAAT: Nucleic acid amplification test
RT-qPCR: Reverse transcription quantitative polymerase chain reaction
Ct: Cycle threshold

## References

[1] Dong E, Du H, Gardner L (2020). An interactive web-based dashboard to track COVID-19 in real time [published correction appears in Lancet Infect Dis. 2020 Sep;20(9):e215]. Lancet Infect Dis.

[2] Kofman A, Kantor R, Adashi EY (2021). Potential COVID-19 endgame scenarios: eradication, elimination, cohabitation, or conflagration?. JAMA.

[3] Centers for Disease Control and Prevention. Interim Guidance for Antigen Testing for SARS-CoV-2. https://www.cdc.gov/coronavirus/2019-ncov/lab/resources/antigen-tests-guidelines.html. Accessed 10 Oct 2021.

[4] Henderson DK, Weber DJ, Babcock H (2021). The perplexing problem of persistently PCR-positive personnel. Infect Control Hosp Epidemiol.

[5] Centers for Disease Control and Prevention. Overview of Testing for SARS-CoV-2, the virus that causes COVID-19. https://www.cdc.gov/coronavirus/2019-ncov/hcp/testing-overview.html. Accessed 14 Feb 2021.

[6] Esbin MN, Whitney ON, Chong S, Maurer A, Darzacq X, Tjian R (2020). Overcoming the bottleneck to widespread testing: a rapid review of nucleic acid testing approaches for COVID-19 detection. RNA.

[7] COVID-19 Test Directory. https://www.finddx.org/tools-and-resources/dxconnect/test-directories/covid-19-test-directory. Accessed 17 Nov 2022.

[8] Abbott. BinaxNOW COVID-19 Ag Card Home Test – Instructions for Use, version 3.4. https://www.fda.gov/media/144574/download. Accessed 23 Aug 2021.

[9] Pilarowski G, Lebel P, Sunshine S (2021). Performance characteristics of a rapid severe acute respiratory syndrome Coronavirus 2 antigen detection assay at a public plaza testing site in San Francisco. J Infect Dis.

[10] Pollock NR, Jacobs JR, Tran K (2021). Performance and implementation evaluation of the Abbott BinaxNOW rapid antigen test in a high-throughput drive-through community testing site in Massachusetts. J Clin Microbiol.

[11] Perchetti GA, Huang ML, Mills MG, Jerome KR, Greninger AL (2021). Analytical sensitivity of the Abbott BinaxNOW COVID-19 Ag Card. J Clin Microbiol.

[12] Abbott RealTime SARS-CoV-2 – Instructions for Use. https://www.fda.gov/media/136258/download. Accessed 19 Nov 2022.

[13] SARS-CoV-2 Assay (Panther Fusion System^®^) – Instructions for Use. https://www.fda.gov/media/136156/download. Accessed 19 Nov 2022.

[14] Centers for Disease Control and Prevention. Ending Isolation and Precautions for People with COVID-19: Interim Guidance. https://www.cdc.gov/coronavirus/2019-ncov/hcp/duration-isolation.html. Accessed 20 Feb 2022.

[15] Singanayagam A, Patel M, Charlett A (2020). Duration of infectiousness and correlation with RT-PCR cycle threshold values in cases of COVID-19, England, January to May 2020. Euro Surveill.

[16] Rao SN, Manissero D, Steele VR, Pareja J (2020). A systematic review of the clinical utility of cycle threshold values in the context of COVID-19. Infect Dis Ther.

[17] La Scola B, Le Bideau M, Andreani J (2020). Viral RNA load as determined by cell culture as a management tool for discharge of SARS-CoV-2 patients from infectious disease wards. Eur J Clin Microbiol Infect Dis.

[18] Fenollar F, Bouam A, Ballouche M (2021). Evaluation of the Panbio COVID-19 rapid antigen detection test device for the screening of patients with COVID-19. J Clin Microbiol.

[19] Carbonell-Sahuquillo S, Lázaro-Carreño MI, Camacho J (2021). Evaluation of a rapid antigen detection test (Panbio™ COVID-19 Ag Rapid Test Device) as a point-of-care diagnostic tool for COVID-19 in a pediatric emergency department. J Med Virol.

[20] Albert E, Torres I, Bueno F (2021). Field evaluation of a rapid antigen test (Panbio™ COVID-19 Ag Rapid Test Device) for COVID-19 diagnosis in primary healthcare centres. Clin Microbiol Infect.

[21] Kuo P, Realegeno S, Pride DT (2021). Comparison of two nucleic acid amplification tests (NAATs) and two antigen tests for detection of SARS-COV-2 from upper repiratory specimens. J Clin Virol Plus.

[22] Li N, Wang X, Lv T (2020). Prolonged SARS-CoV-2 RNA shedding: Not a rare phenomenon. J Med Virol.

